# High Performance Bacteria Anchored by Nanoclay to Boost Straw Degradation

**DOI:** 10.3390/ma12071148

**Published:** 2019-04-09

**Authors:** Minghao Li, Caiguo Tang, Xue Chen, Shengwei Huang, Weiwei Zhao, Dongqing Cai, Zhengyan Wu, Lifang Wu

**Affiliations:** 1 Key Laboratory of High Magnetic Field and Ion Beam Physical Biology, Hefei Institutes of Physical Science, Chinese Academy of Sciences, Hefei 230031, China; limh@ipp.ac.cn (M.L.); cgtang@mail.ustc.edu.cn (C.T.); lmh401@126.com (X.C.); swhuang@ipp.ac.cn (S.H.); annyzhao@ipp.ac.cn (W.Z.); 2 School of Life Sciences, University of Science and Technology of China, Hefei 230026, China; 3 Key Laboratory of Environmental Toxicology and Pollution Control Technology of Anhui Province, Hefei Institutes of Physical Science, Chinese Academy of Sciences, Hefei 230031, China

**Keywords:** bacterial mixture, attapulgite, nanobiosystem, straw returning, degradation

## Abstract

Generally, crop straw degrades slowly in soil, which is unfavorable for tillage and next crop growth. Thus, it is important to develop a promising technology to boost degradation of straw. Herein, a nanobiosystem has been developed by loading bacterial mixture in nanostructured attapulgite (ATP) and using it as a straw returning agent (SRA). Therein, ATP could effectively anchor bacteria to the surface of straw and greatly facilitate the adhesion and growth of bacteria. Consequently, this technology could effectively accelerate the degradation and transformation of straw into nutrients, including nitrogen (N), phosphorus (P), potassium (K), and organic matters (OM). Pot and field tests indicated that SRA displayed significant positive effects on the growth of the next crop. Importantly, SRA could effectively decrease greenhouse gas emissions from farmland, which is beneficial for the environment. Therefore, this work provides a facile and promising method to facilitate the degradation of straw, which might have a potential application value.

## 1. Introduction

In recent years, in accordance with the fast development of agriculture and increase in crop yields, increasing quantities of crop straw have been generated. In many regions, such as the USA, UK, and China, the usual strategy of crop straw treatment is to return them to the soil after grinding [1,2]. Returning straw to soil is an important route to balance C loss from soil and supplement organic matters (OM) to soil [3,4]. However, straw generally degrades slowly, resulting in difficult tillage, a negative effect on the growth of the succeeding crop, and increased emission of greenhouse gases [5,6,7]. It was reported that straw returning increased CH_4_ emission by 110.7% in rice paddies, and N_2_O by 8.3% in upland soil [8]. As such, these negative effects resulting from slow degradation of straw became one of the main restrictions for large scale applications of straw return. Hence, it is critical to develop promising technologies to promote the degradation of crop straws.

Several approaches of speeding up straw degradation have been explored in recent years, including physical grinding [9,10,11], deep ploughing [12], and decomposition agents [13,14,15]. Physical grinding and deep ploughing were difficult to apply at a large scale because of their high energy consumption and cost. Decomposition agents are attracting more and more attention attributed to its low cost, easy operation, and environmentally friendly properties. Nevertheless, traditional decomposition agents possessed low adhesion, weak colonization, and low activity, which greatly restricted its application. Therefore, it is critical to find a way to increase the adhesion of decomposition agents and promote the application of straw returning. 

Attapulgite ((Mg,Al)_4_(Si)_8_(O,OH,H_2_O)_26_·nH_2_O) (ATP) is a kind of natural crystalline hydrated magnesium aluminum silicate, consisting of nanorods approximately 800–2000 nm in length and 30–40 nm in width [16,17,18]. Naturally, the nanorods crosslink with each other to form a nanonetwork structure with high porosity. ATP has been widely used in a variety of applications, such as adsorbent [19,20], carrier [21,22], and construction materials, because of its large specific surface area and high surface activity [23]. 

ATP was used as a carrier to load bacterial mixture (BM) (*Citrobacter freundii*, *Arthrobacter woluwensis*, and *Bacillus licheniformis*), obtaining a nanobiosystem that was used as a straw returning agent (SRA). The promotion effects of ATP on the adhesion and growth of bacteria on the surface of wheat straw were investigated. Additionally, the influences of SRA on the degradation of wheat straw, growth of the maize, and the emission of greenhouse gas were also studied. Moreover, the morphology of SRA on the straw was observed to elucidate the mechanism. Therefore, this work provides a low-cost, facile, and environmentally friendly method to promote the degradation of straws, which has a huge application value in the development of sustainable agriculture all over the world.

## 2. Materials and Methods

### 2.1. Site Description and Materials

The study site (31°53′ N, 117°10′ E; 27 m above sea level) was located on the experimental farm of Hefei Institutes of Physical Science, Chinese Academy of Sciences, China. The primary soil in the area is classified as a yellow clunamon soil, with 15.30 g·kg^−1^ organic matter, 0.13% total N, 74.30 mg·kg^−1^ available P, and 2.08% total K in the upper 0–20 cm of soil profile. 

Natural ATP powder (100–200 mesh, purity of 95%) was purchased from Mingmei Co., Ltd. (Anhui, China). We isolated bacteria (*C. freundii*, *A. woluwensis*, and *B. licheniformis*) and then transformed them to powders (100–200 mesh) by freeze drying and grinding. Maize seeds were purchased from Fengle Seed Co., Ltd. (Hefei, China). Deionized water was used in all the experiments except the pot experiment. Soil used in the pot experiment was taken from Dongpu Island (Hefei, China). 

### 2.2. Preparation of SRA

*C. freundii*, *A. woluwensis*, and *B. licheniformis* were isolated from the gut of root-feeding larvae *Holotrichia parallela* and found to possess cellulolytic activity in our previous studies [24,25]. For constructing bacterial mixture, *C. freundii*, *A. woluwensis*, and *B. licheniformis* grew in Luria-Bertani (LB) at 30 °C for 20–24 h in a rotary shaker incubator (180 rpm), respectively. Then, the cells were harvested by centrifugation for 20 min at 8000× *g* and dissolved in sterile MilliQ water to obtain a final cell concentration of 10^7^ CFU/mL, respectively. The bacterial strains were added and suspended in the freeze-dried protective agent containing dried skim milk and trehalose. After freezing for 12 h at an ultra-low temperature, the three bacterial strains were freeze-dried for 24 h at −50 °C with a vacuum freeze drier (Beijing Songyuan Huaxing Co., Ltd., Beijing, China). Finally, the three bacteria were evenly mixed with ATP with a weight ratio of 1:2:2:5 to form the SRA. 

### 2.3. Straw Degradation Investigation

SRA aqueous solution (5 mL, 1 g/L) was evenly spayed onto 20 g of wheat straw (variety of Yangmai 158, average length of approximately 5 cm) after harvest, and the resulting straw was placed in a cylindrical nylon net bag (diameter of 15 cm, height of 25 cm). After that, the bag was buried in soil (humidity of 30%) with a depth of 5–10 cm. The bag was dug out after 20, 40, 60, 80, and 100 d. Finally, length and weight of the remaining straw were determined after air drying. Nitrogen (N) content by alkali hydrolyzable method [26], phosphorus (P) by spectrophotometer (Shimadzu, Kyoto, Japan), and potassium (K) by flame emission photometry in the resulting straw were measured, respectively [27]. All experiments were carried out in triplicate.

### 2.4. Pot Experiment

SRA (5 mL, 2 g/L) or BM (5 mL, 1 g/L) aqueous solution was evenly spayed onto wheat straw (20 g, average length of approximately 5 cm) after harvest, and the resulting straw was evenly mixed with 3.5 kg of soil (humidity of 30%). The resulting mixture was then placed in a pot (trapezoidal shape, height of 10 cm, and diameters of 18 cm (top) and 10 cm (bottom)). Subsequently, three maize seeds were put in the soil with a depth of 5 cm. The resulting system was placed in a greenhouse under a temperature of 25 °C, and 50 mL of water was sprayed evenly to the system every three days. At given intervals after seeding, plant height, stem diameter, chlorophyll content, and photosynthetic rate in leaves were measured. All experiments were performed in triplicate.

### 2.5. Pyrosequencing of 16S rRNA Gene

To characterize the bacterial composition and community diversity under different treatments (straw alone, BM, and SRA), soil samples of pot experiment of each treatment were randomly selected for pyrosequencing analysis. The soil total DNA was extracted from 0.25 g of cryopreserved samples by DNeasy PowerSoil Pro Kit (Qiagen, Germantown, MD, USA), according to instructions from the manufacturer. Then the DNA were detected by 1% agarose-gel electrophoresis, and the concentration and purity of the DNA were detected by NanoDrop 2000 Microvolume Spectrophotometers and Fluorometer (Thermo Scientific, Waltham, MA, USA). The sample was frozen at −80 °C until pyrosequencing analysis. Bacterial 16S rRNA gene amplification (Applied Biosystems, Carlsbad, NM, USA) and barcoded pyrosequencing were performed on an Illumina MiSeq instrument (Illumina, San Diego, CA, USA). Briefly, triplicate polymerase chain reaction (PCR) products of each sample were pooled, purified, and quantified using a NanoDrop 2000 Microvolume Spectrophotometers and Fluorometer (Thermo Scientific, Waltham, MA, USA) after PCR amplification of the 16S V_4_ region using the 515F primer (Sangon Biotech Co., Ltd, Shanghai, China) and barcoded versions of 806R (Sangon Biotech Co., Ltd, Shanghai, China). Equimolar concentrations of the purified amplicons were pooled together and subjected to paired-end multiplex sequencing using an Illumina MiSeq instrument At Majorbio Bio-pharm Technology Co., Ltd. (Shanghai, China). For raw data analysis, only illumine reads over 175 nt were analyzed (97% of the obtained high-quality reads). Operational taxonomic units (OTUs) were defined at 97% nucleotide similarity. Taxonomic classification was done using the Bayesian classifier provided by the mothur software package (University of Michigan Medical School, Ann Arbor, MI, USA) [28]. The relative abundance of a given phylogenetic group was set as the number of sequences affiliated with that group divided by the total number of sequences per sample. 

### 2.6. Gas Emission Investigation

**Figure 1:**
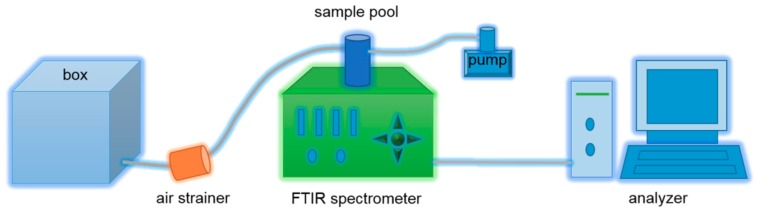
Schematic diagram of greenhouse gas detection system.

SRA (20 mL/m^2^, 2 g/L) or BM (20 mL/m^2^, 1 g/L) aqueous solution was evenly sprayed onto wheat straw (average length of approximately 5 cm) in a field (100 m^2^) after harvest, and the resulting straw was returned to soil (depth of 0–10 cm, humidity of 20–40%) using a rotary tiller (Yituo Co., Ltd, Luoyang, Henan, China). A cubic glass box (1 cubic meter) was then placed in the field with the open side downward (Figure 1), and the gas concentrations of CO_2_, CH_4_, and N_2_O in the box were determined after 60 d. Each processing test was repeated three times. 

### 2.7. Determination of Chlorophyll and Photosynthetic Efficiency

The chlorophyll content and photosynthetic rate in maize leaves were determined by a chlorophyll meter (Konica Minolta Investment Ltd., Tokyo, Japan) and photosynthetic rate apparatus (Li-6400, LI-COR, Lincoln, NE, USA), respectively. All the above indexes were determined from the top leaf of the plant, nine strains were selected for each treatment and the average value was taken.

### 2.8. Chemical Characterization Analysis

The morphology of the straw samples was measured by a scanning electron microscope (SEM) (Sirion 200, FEI Co., Hillsboro, OR, USA). The structure and interaction were analyzed by an X-ray diffractometer (X’Pert, Philips Co., Amsterdam, The Netherlands) with Cu radiation (40 kV, 200 mA, λ = 1.541867 Å, 2θ = 3° to 70°, scanning speed = 8 °/min, step size = 0.0200°). The samples were washed with sterile water several times to remove the bacteria from the surface of the straw and dried in a constant temperature drying oven (HASUC, Shanghai, China) until the quality was constant. The dried straw samples were mixed with KBr and ground to power, where the sample content in KBr was 0.5%, then characterized by a Fourier transform infrared (FTIR) spectroscopy (iS10, Nicolet Co., Madison, WI, USA) at ambient temperature. 32 scans were recorded in a 400–4000 cm^−1^ spectral range at a resolution of 4 cm^−1^ for each sample. The thermal gravimetric analysis (TG) and differential thermal analysis (DT) were performed by a thermogravimetric analyzer (Q5000IR, TA Co., Wilmington, DE, USA) at a scan rate of 10 °C /min from 40 to 800 °C in oxygen or nitrogen. 

### 2.9. Statistical Analyses

A two-way analysis of variance (ANOVA) was performed to determine the effects of the treatments. Significant differences in the means of three replicates among all the treatments were considered at *P* < 0.05 using Fisher’s least significant different (LSD). Statistical analysis was performed using SPSS 16.0 software for Windows.

## 3. Results 

### 3.1. Effect of SRA on Degradation of Straw

**Figure 2:**
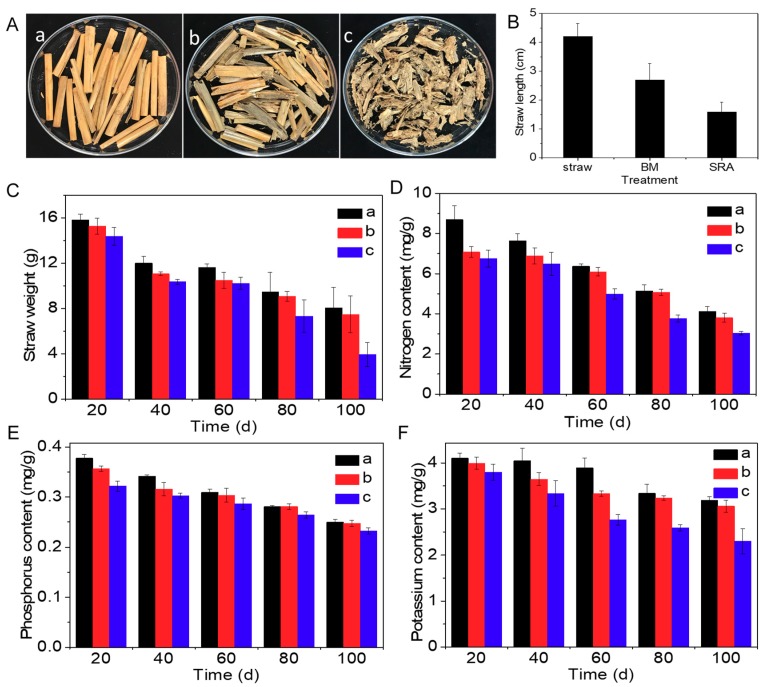
(**A**) Digital photographs of (**a**) wheat straw alone and those treated by (**b**) bacterial mixture (BM) and (**c**) straw returning agent (SRA) for 100 d. (**B**–**F**) Length, weight, nitrogen, phosphorus, and potassium contents of the resulting straws.

Wheat straw mainly consisted of cellulose, hemicellulose, lignin, and inorganic substances and thus degraded slowly in soil, which greatly limited tillage and growth of next crop. In order to evaluate the promotion effect of SRA on the degradation of straw, the dynamic influence of SRA on the length, weight, nitrogen, phosphorus, and potassium contents of wheat straw was investigated compared with wheat straw alone and BM. As shown in Figure 2Aa,b, after being buried in soil for 100 d, the wheat straws alone were still yellow, integrate and compact, displaying little change compared with the original straw. While a part of the BM-treated straws became gray, incomplete, and loose (Figure 2Ab), suggesting that BM could accelerate the degradation of straw to a certain extent. As for the SRA-treated straws, all of them became gray, fractured, loose, and short (Figure 2Ac), demonstrating that SRA possessed a significantly higher promotion effect on the degradation of straw compared with BM. As illustrated in Figure 1B, on the 100th day, the average lengths of straw alone, BM-treated, and SRA-treated were 4.2, 2.7, and 1.6 cm, respectively, indicating their degradation efficiency order: SRA-treated > BM-treated > straw alone. Additionally, it could be clearly seen in Figure 2C–F that all the four parameters (weights, nitrogen, phosphorus, and potassium contents) of those three kinds of straws decreased with time, wherein those of SRA-treated straw decreased the fastest, which also proved the excellent effect of SRA on the degradation of straw. On one hand, the smaller and looser straws induced by SRA were beneficial for the tillage. On the other hand, the degraded part of the straw was probably transformed to small substances including organic matters, nitrogen, phosphorus, and potassium, which could partly exist in soil and be used as the nutrients for the next crop.

### 3.2. Effect of SRA on Growth of Maize

**Figure 3:**
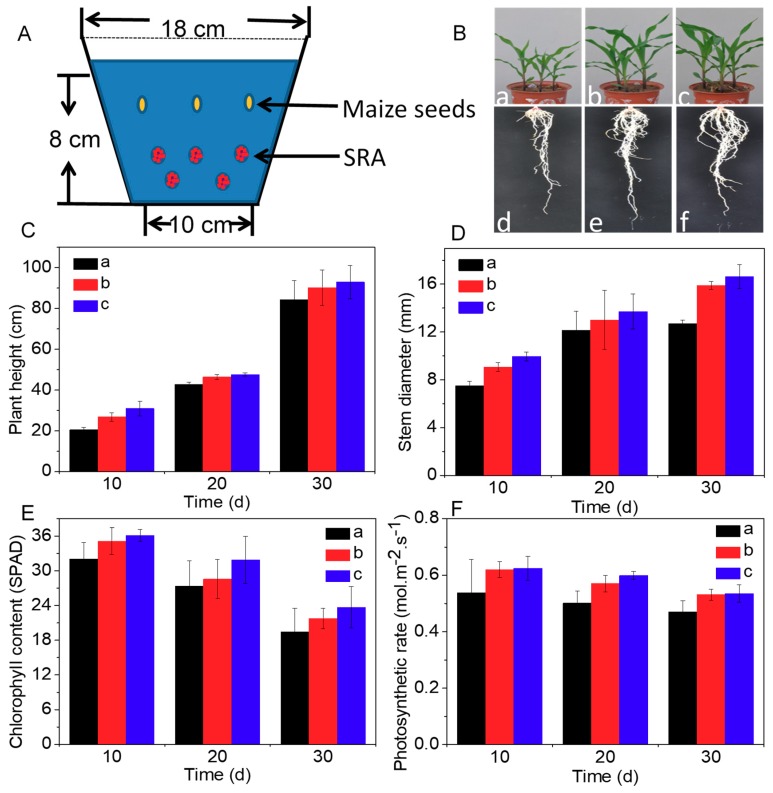
(**A**) Schematic diagram of the pot experiment system. (**B**) Digital photographs of maize in pots after returning of (**a**) wheat straw alone and those treated by (**b**) BM and (**c**) SRA for 30 d, and (**d**–**f**) their roots. (**C**–**F**) Plant height, stem diameter, chlorophyll content and photosynthetic rate in leaves of those maize.

In order to understand the effect of SRA on maize growth, a pot experiment was carried out, wherein three maize seeds were placed in soil with wheat straw alone and those treated by BM and SRA, respectively (Figure 3A). As illustrated in Figure 3B, after seeding for 30 d, the growth and roots of maize in those three kinds of soil displayed an order: SRA-treated > BM-treated > straw alone, which was in accordance with the preceding degradation result. Additionally, the plant height, stem diameter, chlorophyll content, and photosynthetic rate in the leaves of the maize displayed a similar trend (Figure 3C–F). Therein, the plant height and stem diameter of the maize increased with time (Figure 3C,D). Notably, from the 20th to 30th day after seeding, the stem diameter of the maize treated by SRA and BM increased significantly faster compared with maize without treatment (Figure 3D). In addition, the chlorophyll content and photosynthetic rate of those maize leaves decreased with time, which was consistent with the growth characteristic of maize (Figure 3E,F) [29]. These results indicated that returning SRA-treated straw could efficiently favor the growth of maize compared with BM and straw alone, which was attributed to the significant promotion effect of SRA on the degradation of straw and transformation of the degraded straw to abundant nutrients for maize.

**Figure 4:**
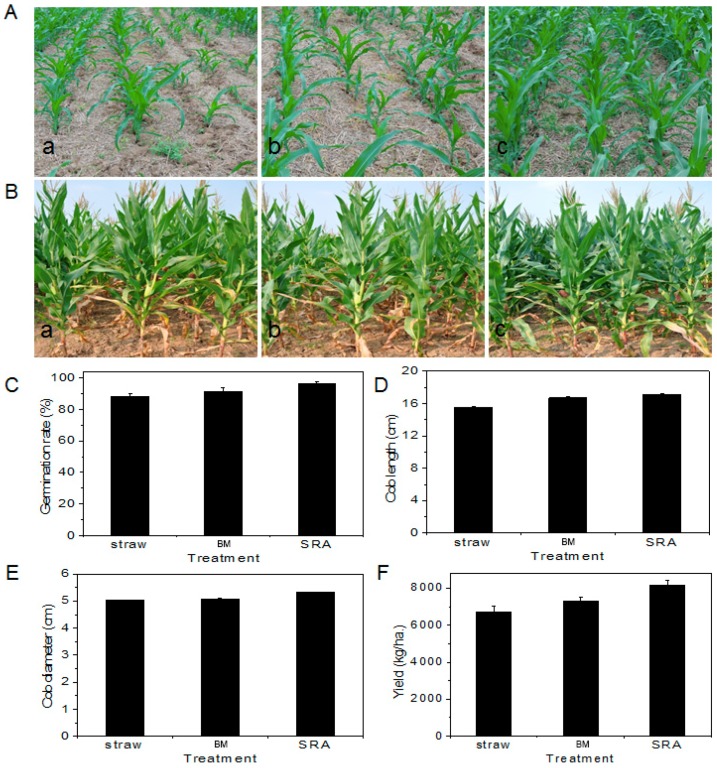
Digital photographs after seeding for 20 (**A**) and 80 d (**B**), respectively, of wheat straw alone (**a**) and those treated by (**b**) BM and (**c**) SRA. (**C**) germination rate, (**D**) Cob length, (**E**) cob diameter, and (**F**) yield of maize in fields (100 m^2^).

A field test was also performed to further obtain the effect of SRA on the germination, growth, and yield of maize. As shown in Figure 4A, after seeding for 20 d, BM-treated maize showed better germination and growth compared with straw alone, and SRA-treated maize showed significantly better germination and growth compared with BM-treated maize. After seeding for 80 d, the SRA-treated maize possessed more cobs than the BM-treated and straw alone (Figure 4B). Additionally, it could be seen clearly in Figure 4C–E that the germination, cob length, and cob diameter of maize displayed the same order: SRA-treated > BM-treated > straw alone, which corresponded to the preceding degradation and pot test results. Importantly, the yield of maize also illustrated a similar order: SRA-treated (8165 Kg/ha) > BM-treated (7295 Kg/ha) > straw alone (6705 Kg/ha) (Figure 4F). Notably, the SRA-treated maize dramatically increased the cob diameter compared with the BM-treated maize (Figure 4E). These results provide further evidence for the positive effect of SRA on the growth of maize.

### 3.3. Effect of SRA on the Emission of Greenhouse Gas

**Figure 5:**
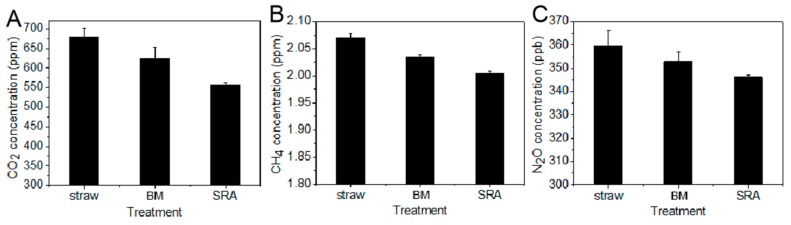
(**A**–**C**) CO_2_, CH_4_, and N_2_O concentrations from maize fields after returning of wheat straw alone and those treated by BM and SRA for 60 days, respectively.

Wheat straw returning could significantly stimulate CH_4_ emission [30,31]. Soil CH_4_ emission is primarily regulated by the availability of C substrates in the soil, and wheat straw could provide available C sources as organic materials to support the growth of methanogenic populations under anaerobic conditions [32]. Previous studies indicate that the effect of straw returning on N_2_O emission is different. Xia et al. found that long-term straw incorporation can reduce N_2_O emissions compared to no straw return [31]. In contrast, other results demonstrated that straw returning could increase N_2_O emission by 15–39% [33]. We obtained the influence of SRA on the emission of greenhouse gas including CO_2_, CH_4_, and N_2_O from a field after treatment for 60 days. As demonstrated in Figure 5A–C, the BM treatment could decrease the emission of CO_2_, CH_4_, and N_2_O in a certain degree compared with straw alone, and SRA could dramatically decrease the emission of those gases compared with BM. This result was in accordance with the preceding results, indicating that the decreased gas emission after SRA treatment probably resulted from the promotion effect of SRA on the degradation of straw. That is to say, SRA could effectively transform the degraded straw to nutrients rather than gas, which was favorable for improving soil quality and reducing its contribution to global warming. 

### 3.4. Effect of SRA on the Soil Microbial Community

**Figure 6:**
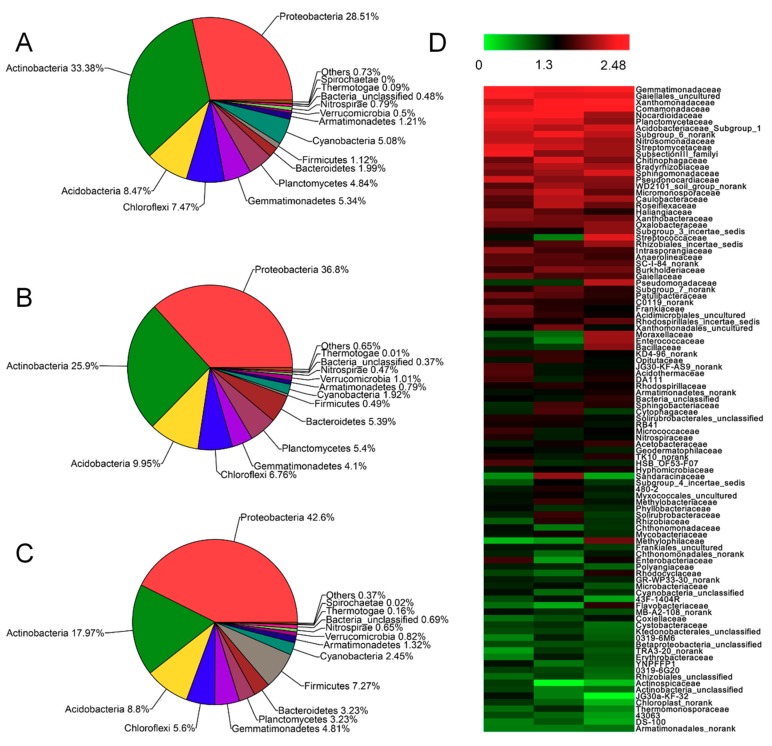
(**A**–**C**) Microbial community in soil (pot) after returning of (**A**) wheat straw alone and those treated by (**B**) BM and (**C**) SRA for 30 d. (**D**). Heatmap comparison of the abundance of the top 100 Families across samples.

Soil microorganisms are important drivers involved in the process of degradation, transformation, and utilization of crop straw [34]. Some studies report that the application of cellulose-decomposing bacteria could effectively accelerate the straw decomposition process and perfect soil nutrient availability [35]. In this study, the influence of SRA on the microbial community in soil was investigated. Sequence numbers ranged from 31,330 to 32,295 sequences per sample. To normalize data sets, a random subsample of 31,330 high quality sequences per fecal sample was used for all analyses. As shown in Figure 6A–C, after treatments of BM and SRA for 30 d, the microbial communities changed to different degrees. Notably, BM could increase the amount of Proteobacteria from 28.51% to 36.8% compared with wheat straw alone, and SRA could further increase the amount of Proteobacteria from 36.8% to 42.6%. In fact, the increased amount of Proteobacteria, a kind of bacteria that can facilitate the degradation of straw, greatly favored the promotion effect of SRA on the degradation of straw and transformation of the degraded straw to nutrients. Moreover, differences were found in the relative abundance of top 44 class, family, and genera between different treatments (Figure 6D). Gaiellales_uncultured, Gemmatimonadaceae, Nocardioidaceae, Streptomycetaceae, Planctomycetaceae, Comamonadaceae, Acidobacteriaceae_Subgroup_1, and Pseudonocardiaceae were the dominant family in the straw alone, while Nocardioidaceae, Comamonadaceae, Xanthomonadaceae, Gemmatimonadaceae, Chitinophagaceae, Planctomycetaceae, and Streptomycetaceae were the dominant family in the BM. However, in the SRA, Xanthomonadaceae, Gemmatimonadaceae, Comamonadaceae, Gaiellales_uncultured, Streptomycetaceae, and Sphingomonadaceae were the dominant family.

### 3.5. Structure and Interaction Analyses

**Figure 7:**
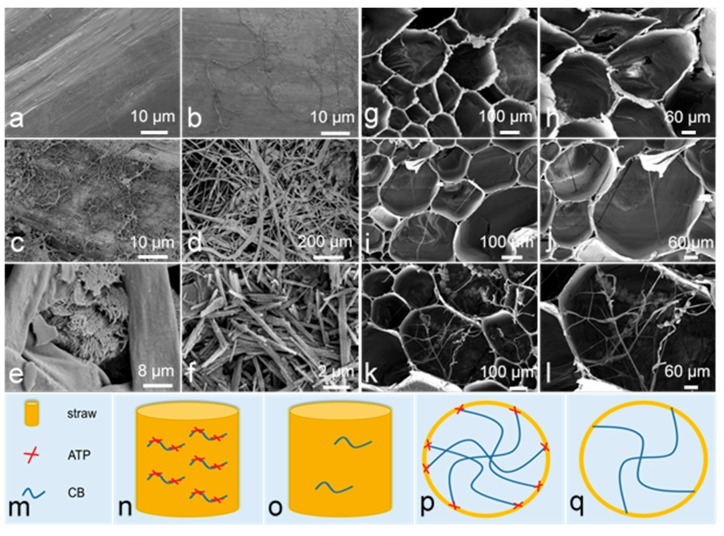
SEM images of (**a**) longitudinal section of wheat straw, (**b**) BM on longitudinal section of wheat straw, (**c**–**e**) SRA on longitudinal section of wheat straw, (**f**) attapulgite (ATP), (**g**,**h**) cross section of wheat straw, (**i**,**j**) BM in cross section of wheat straw, and (**k**,**l**) SRA in cross section of wheat straw. Schematic diagram of anchoring effect of SRA or BM on longitudinal (**n**,**o**) and cross sections (**p**,**q**) of wheat straw with notes (**m**).

In order to elucidate the mechanism of SRA on the degradation of straw, the morphologies of SRA on the longitudinal and cross sections of wheat straw were observed. As shown in Figure 7a, no obvious bacterium was found on the longitudinal section of wheat straw. While, after treatment of BM for 100 d, a number of bacteria appeared (Figure 7b). Furthermore, after treatment of SRA for 100 d, a large number of bacteria existed evenly on the longitudinal section (Figure 7c). Interestingly, as shown in Figure 7d–f, a great number of ATP nanonetworks consisting of abundant nanorods adhered on the section and acted as anchors for bacteria to enhance their adhesion. As for the cross sections of straw alone and treated by BM for 100 d (Figure 7g–l), rather few bacteria were found on the sections, while a great number of bacteria appeared after treatment of SRA, indicating that SRA could efficiently increase the number of bacteria. Noteworthily, ATP nanonetworks also existed between bacteria and the inner surface of straw, acting as anchors to boost the adhesion and growth of bacteria. In fact, ATP nanorods possessed plenty of –OH and –OH_2_ on the surface, thus hydrogen bonds (H-bonds) tended to form between ATP and straw or BM, promoting the adhesion and growth of BM on the straw surface (Figure 7m–q), which was probably the dominant mechanism for the promotion of straw degradation. 

**Figure 8:**
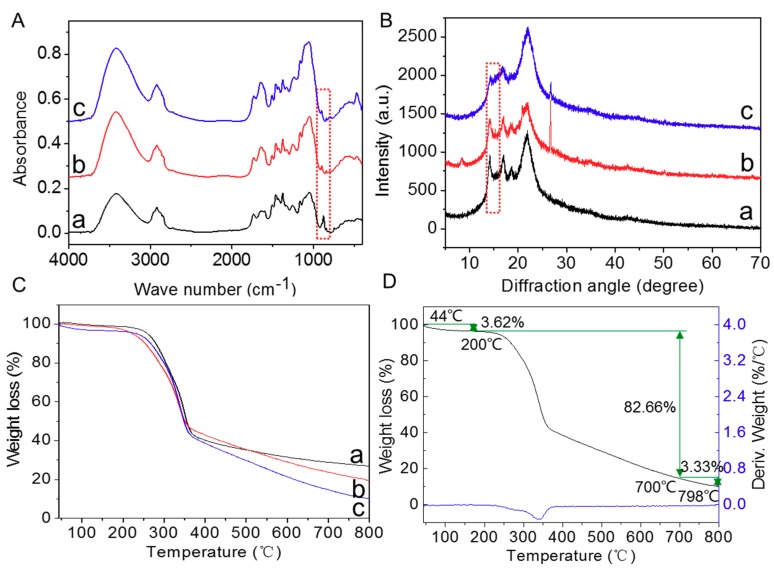
(**A**) Fourier transform infrared (FTIR), (**B**) XRD, and (**C**) thermal gravimetric (TG) spectra of (**a**) wheat straw, (**b**) BM-treated wheat straw, and (**c**) SRA-treated wheat straw after treatment for 100 d, (**D**) TG (black) and differential thermal (DT) (blue) curves of SRA-treated wheat straw.

To further investigate the mechanism of SRA, FTIR measurements were performed. As shown in Figure 8A, after BM treatment for 100 d, the original peak at 876 cm^−1^ corresponding to cellulose, galactose, mannose, Arabia sugar, and polysaccharide in the spectrum of straw alone left-shifted to 898 cm^−1^ and became weak, indicating that BM promoted the degradation of those substances. Additionally, after SRA treatment for 100 d, the relative strength of the peak at 898 cm^−1^ further weakened compared with BM, suggesting that SRA displayed a higher promotion performance on the degradation of those substances than BM. The result demonstrated that SRA mainly promoted the degradation of cellulose, galactose, mannose, Arabia sugar, and polysaccharide in wheat straw. In addition, XRD analysis was also performed to investigate the influence of SRA on the crystal structure of straw. As illustrated in Figure 8B, the peak at 15° became significantly weak compared with BM and straw alone, proving that SRA greatly promoted the damage of the crystal structure of straw. 

TG analysis was conducted to evaluate the thermal stability of straw with different treatments. As shown in Figure 8C and D, three distinct regions of weight loss were observed in the TG curve. The first region (45–200 °C) most likely corresponded to the loss of water. The second region (200–700 °C) was attributed to the decomposition of straw at high temperature. The third region (>700 °C) was attributed to the end of straw decomposition. It could be seen clearly in Figure 8D that, at 800 °C, the weight loss of those straws displayed an order: SRA-treated (82.66%) > BM-treated (76.03%) > straw alone (71.63%), which was consistent with their degradation abilities. This result suggested that SRA possessed a higher degradation ability on straw and thus the resulting straw displayed a lower thermal stability compared with BM and straw alone. 

**Figure 9:**
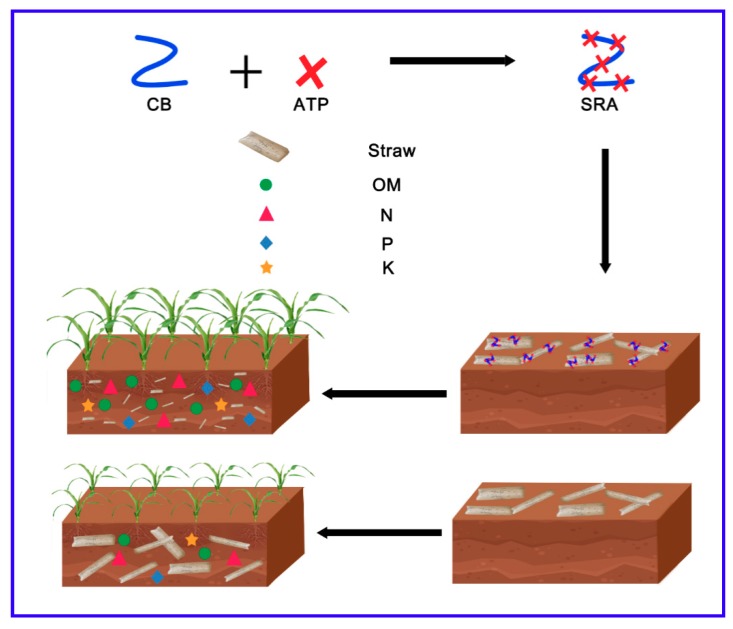
Schematic illustration of fabrication and application of SRA in a field.

In large-scale practice (Figure 9), crop straw is cut into abundant small pieces with a length of 5–10 cm and evenly distributed on the surface of soil in a field after harvest. Then SRA aqueous solution with a certain concentration is sprayed evenly onto the surface of straw. After that, the resulting straw is returned to soil with depth of 0–10 cm using a rotary tiller. Finally, the SRA could effectively accelerate the degradation of straw, transform part of it to nutrients, and thus promote the growth of the next crop. As such, SRA could be used conveniently in agriculture and thus has a huge application prospect. 

## 4. Conclusions

In this work, SRA was fabricated through incorporation of ATP and BM, wherein ATP nanonetworks acted as an ideal carrier for BM, anchored BM on the surface of straw, enhanced the adhesion, promoted the growth of BM, and thus accelerated the degradation of straw and release of nutrients. Thus, this technology could effectively promote the growth of the next crop. Importantly, this technology could also significantly reduce greenhouse gas emission and increase the number of bacteria, which were beneficial for the degradation of straw. This work provides a low-cost and simple approach to accelerate straw degradation and protect the environment, which has promising application prospects.
